# Evaluation of an Element-Tagged Duplex Immunoassay Coupled with Inductively Coupled Plasma Mass Spectrometry Detection: A Further Study for the Application of the New Assay in Clinical Laboratory

**DOI:** 10.3390/molecules25225370

**Published:** 2020-11-17

**Authors:** Wencan Jiang, Gongwei Sun, Wenbin Cui, Shasha Men, Miao Jing, Danna Pu, Sichun Zhang, Xiaozhou Yuan, Xinrong Zhang, Chengbin Wang

**Affiliations:** 1 Department of Clinical Laboratory Medicine, Chinese People’s Liberation Army General Hospital & Postgraduate Medical School, Beijing 100853, China; jiangwencan2014@126.com (W.J.); menshasha2006@126.com (S.M.); pudanna301@163.com (D.P.); yuanxiaozhou123@sina.com (X.Y.); 2 Beijing Key Laboratory for Microanalytical Methods and Instrumentation, Department of Chemistry, Tsinghua University, Beijing 100084, China; bjtasi@163.com (G.S.); sczhang@mail.tsinghua.edu.cn (S.Z.); 3 Chromatography & Mass Spectrometry Thermo Fisher Scientific, China Commercial, Beijing 100853, China; wenbin.cui@thermofisher.com (W.C.); miao.jing@thermofisher.com (M.J.); 4 Department of Chemistry, Tsinghua University, Beijing 100084, China; 5 Fuxing Road 28, Haidian district, Beijing 100853, China

**Keywords:** ICP-MS-based duplex immunoassay, CEA, AFP, evaluation, performance, clinical sample detection

## Abstract

Background: Element-tagged immunoassay coupled with inductively coupled plasma mass spectrometry (ICP-MS) detection has the potential to revolutionize immunoassay analysis for multiplex detection. However, a further study referring to the standard evaluation and clinical sample verification is needed to ensure its reliability for simultaneous analysis in clinical laboratories. Methods: Carcinoembryonic antigen (CEA) and α-fetoprotein (AFP) were chosen for the duplex immunoassay. The performance of the assay was evaluated according to guidelines from the Clinical and Laboratory Standards Institute (CLSI). Moreover, reference intervals (RIs) of CEA and AFP were established. At last, 329 clinical samples were analyzed by the proposed method and results were compared with those obtained with electrochemiluminescent immunoassay (ECLIA) method. Results: The measurement range of the assay was 2–940 ng/mL for CEA and 1.5–1000 ng/mL for AFP, with a detection limit of 0.94 ng/mL and 0.34 ng/mL, respectively. The inter-assay and intra-assay imprecision were all less than 6.58% and 10.62%, respectively. The RI of CEA and AFP was 0–3.84 ng/mL and 0–9.94 ng/mL, respectively. Regarding to clinical sample detection, no significant difference was observed between the proposed duplex assay and the ECLIA method. Conclusions: The ICP-MS-based duplex immunoassay was successfully developed and the analytical performance fully proved clinical applicability. Well, this could be different with other analytes.

## 1. Introduction

Biomarker quantification is of great importance in the diagnosis of cancer and other diseases, since it can reflect the occurrence and development of diseases and monitor the response to treatment [1,2,3]. Currently, antibody and antigen specific binding based immunoassays are commonly used tools for the target quantification of biomarkers [4]. Looking back, it is known that immunoassays have been used since 1959, when the technology of radioimmunoassay (RIA) was first reported for the detection of insulin [5]. However, the problems of waste disposal, limited stability and radioisotopes pollution restricted the use of the RIA method [6]. Regarding the problem, a variety of sophisticated non-radioactive immunoassays had gradually been proposed, including enzyme linked immunosorbent assay (ELISA), fluorescence immunoassay (FIA), time resolved fluorescence immunoassay (TAFIA), chemiluminescent immunoassay (CLIA), and electrochemiluminescent immunoassay (ECLIA) [4,7,8,9,10,11,12]. However, these conventional immunoassay methods are often significantly limited for simultaneous multianalyte quantification. To detect several biomarkers simultaneously for a quick diagnostic statement, some multianalyte assays were proposed, such as quantum dots fluorescence immunoassay, up-converting phosphor based immunoassay, etc. [13,14]. In comparison to conventional methods, these technologies have the advantages of multiplexing, time reduction, smaller reaction quantities and automation [15]. However, no matter what labels (e.g., radioisotope, fluorescent dye or enzyme) are used, overlap of signals occurs, which limits their popularization and application in clinical multiplex analysis [16,17].

In comparison with other immunoassays, inductively coupled plasma mass spectrometry (ICP-MS) and element-tagged based immunoassay is a powerful technique for the simultaneous determination of multiple biomolecules, since it could avoid the drawback of spectral overlap [18]. In this technique, multiple rare earth elements (REEs) or their stable isotopes were labeled on the antibodies, distinguished by the mass/charge ratio of the elements. Following the first report of the ICP-MS-based immunoassay for the detection of thyroid hormones in 2001 [19,20], a variety of assays were reported for the quantification of medical markers including duplex labels, multiple labels, and even more than 40 labels [20,21,22,23,24,25,26,27,28,29,30]. Advantages of the ICP-MS-based immunoassay include high sensitivity, high stability, high-level multiplex analysis, low matrix effects, and wide dynamic ranges [30]. The implementation of this method will not only promote the application of mass spectrometry in clinical laboratories, but also reduce the cost of testing, and at the same time provide a new alternative in various laboratories. Although the concept has great clinical potential, its clinical implementation requires standard evaluation and clinical detection reference to documents from the clinical and laboratory standards institute (CLSI).

The development of clinical analysis technology is continuing and progressive. In order to ensure the reliability of the application for the ICP-MS-based immunoassay, the verification of simplex, duplex, and multiplex detection should be performed orderly. The verification of the simplex assay has been successfully finished for the detection of carcinoembryonic antigen (CEA) in the initial stage by our group [31]. However, it does not fully represent the feasibility of multiple detection in clinical laboratories. Therefore, the clinical implementation and transformation of multiplex detection for the ICP-MS-based immunoassay requires additional steps, that is, the standard methodological evaluation and clinical sample verification of dual or multiple detection systems referring to CLSI guidelines.

The test of CEA and AFP is a cheap, safe and noninvasive test for assessing tumor status and therapeutic efficacy in cancer patients [32,33]. Analysis of CEA and AFP are widely used in clinical diagnostics; for example, there are nearly 20,000 clinical samples every month in our hospital. Thus, CEA and AFP were chosen to evaluate the ICP-MS-based duplex immunoassay. Firstly, the performance (such as interference, overlap, etc.) of the ICP-MS analyzer for the detection of the used element were evaluated. Secondly, following the establishment and optimization of the ICP-MS-based duplex immunoassay, the evaluation of the proposed method was carried out and the reference intervals (RIs) for CEA and AFP were established with the guidance of the CLSI guidelines. Finally, 329 clinical samples were collected and measured using the proposed method, and the results were compared with the ECLIA method.

## 2. Results

### 2.1. Evaluation of the ICP-MS Instrument

As is shown in Supplemental Figure S1, there was no significant difference between the different interference groups for Eu^3+^, although there was significant difference between the different interference groups for Sm^3+^, when the element Re was selected as the internal control element. According to the Eu/Re value and Sm/Re value for the repeated tests (50 times), the CVs were all calculated to be less than 5.00%. With regard to the source of the signal, there was no significant difference between the signal of the blank and signals from serum, PBS buffer, water, and antibodies (Supplemental Figure S2).

**Figure 1:**
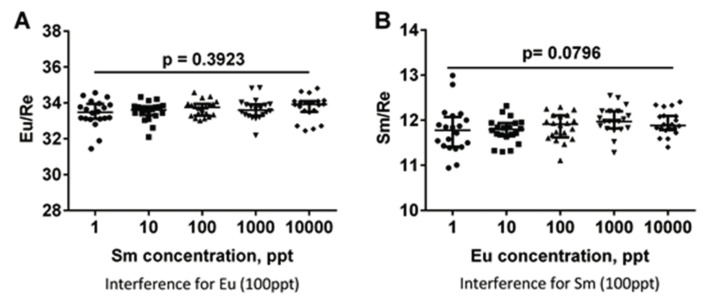
Evaluation of overlaps between Eu and Sm. (**A**) Interference for the element Eu, there was no significant difference for Eu/Re value between the different concentration groups of Sm; (**B**) Interference for the element Sm, there was no significant difference for Sm/Re value between different concentration groups of Eu.

Regarding the evaluation of overlaps between the element Eu and Sm, as is shown in Figure 1, there was no significant difference for the Eu/Re value between the different concentration groups of Sm, and there was no significant difference for the Sm/Re value between the different concentration groups of Eu. As for the carryover of the analyzer for Sm^3+^, the results (Supplemental Table S2) implied that the carryover of the ICP-MS instrument was less than 0.5%.

### 2.2. Optimization of the Assay

**Figure 2:**
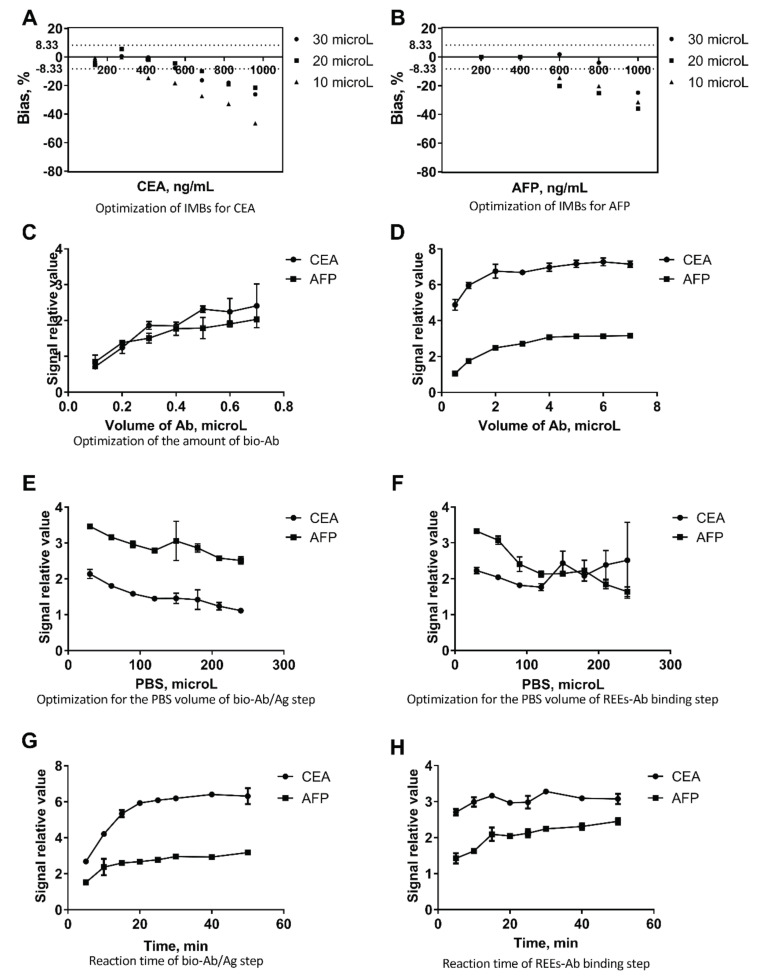
Optimization of the inductively coupled plasma mass spectrometry (ICP-MS) based immunoassay. (**A**) Optimization of the volume of IMBs for carcinoembryonic antigen (CEA), the bias for CEA detection is the smallest when the volume of IMBs is 20 microL; (**B**) Optimization of the volume of IMBs for α-fetoprotein (AFP), the bias for AFP detection is the smallest when the volume of IMBs is 30 microL; (**C**) Optimization of the MMPs and antibody binding ratio, the relative signal value tended to be stable when the volume of bio-AFP and bio-CEA was set at 0.5 microL; (**D**) Optimization for the amount of Eu-labeled CEA antibody and Sm-labeled AFP antibody, 4 microL of Eu-labeled CEA antibody and and 4 microL of Sm-labeled AFP antibody were the most suitable dose for the immunoassay; (**E**) Optimization for the PBS volume of the bio-Ab/Ag step, the optimal volume of the buffer was 30 microL for the IMB-antigen binding step; (**F**) Optimization for the PBS volume of the labeled antibody binding step, the optimal volume of the buffer was 30 microL for the IMB-antigen and labeled antibody binding step; (**G**) Optimization for the reaction time of the bio-Ab/Ag step, the optimal reaction time was 20 min for the IMB-antigen binding step; (**H**) Optimization for the reaction time of the labeled antibody binding step, the optimal reaction time was 10 min for the IMB-antigen and labeled antibody binding step.

The results of assay optimization are shown in Figure 2. The bias for CEA and AFP detection was small when the volume of IMBs for CEA and AFP were 20 microL and 30 microL, respectively. As for the MMPs and antibody binding step, the relative signal value tended to be stable when the volume of bio-AFP and bio-CEA was set at 0.5 microL. With regard to the amount of Eu-labeled CEA antibody and Sm-labeled AFP antibody, 4 microL of Eu-labeled CEA antibody and 4 microL of Sm-labeled AFP antibody were the most suitable doses for the immunoassay. The optimal amount of PBS buffer was 30 μL for both the IMB-antigen binding step and the labeled antibody binding step. From Figure 2H, it seems that the optimal reaction times are 20 and 15 min, respectively (no big difference between 10 and 15 min, though).

### 2.3. Evaluation of Calibration Curves

**Figure 3:**
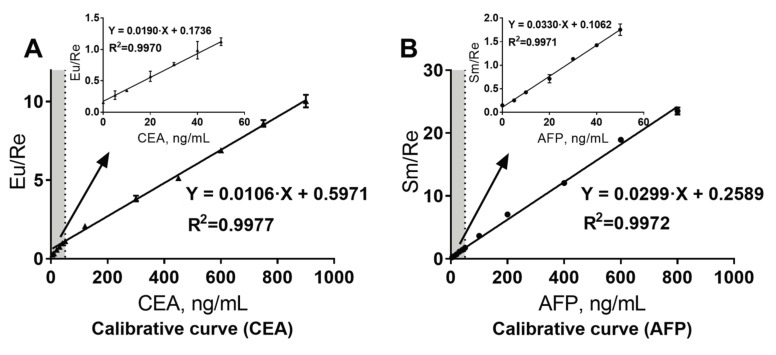
Calibration curve for the immunoassay. (**A**) Calibrative curve of CEA, if the detection signal Eu/Re of a sample was less than 1.1246, the concentration could be calculated using the formula Y = 0.0190·X + 0.1736; if the detection signal Eu/Re of a sample was more than 1.1246, the concentration could be calculated by the formula Y= 0.0106·X + 0.5971; (**B**) Calibrative curve of AFP, if the detection signal Sm/Re of a sample was less than 1.7547, the concentration could be calculated using the formula Y = 0.0330·X + 0.1062; if the detection signal Sm/Re of a sample was more than 1.7547, the concentration could be calculated by the formula Y = 0.0299·X + 0.2589.

The calibration curve of CEA is shown in Figure 3. The LoD of the proposed assay for CEA and AFP are 0.93 ng/mL and 0.34 ng/mL, respectively. The LLoQ of the assay was 2 ng/mL for CEA and 1.5 ng/mL for AFP (Supplemental Table S3). The ULoQ of the immunoassay for CEA and AFP was 940 ng/mL and 1000 ng/mL, respectively (Supplemental Figure S3). As for the allowable dilution ratio and CRR, when the dilution ratio was below 10, the values in Supplementary Tables S4 and S5 are 6.35 and 6.78%, respectively. Thus, the acceptable dilution ratio was 10 for the immunoassay. The upper clinical reportable limit of detection (obtained by multiplying ULoQ by the acceptable dilution ratio) for CEA and AFP was 9400 ng/mL and 10,000 ng/mL, respectively.

**Figure 4:**
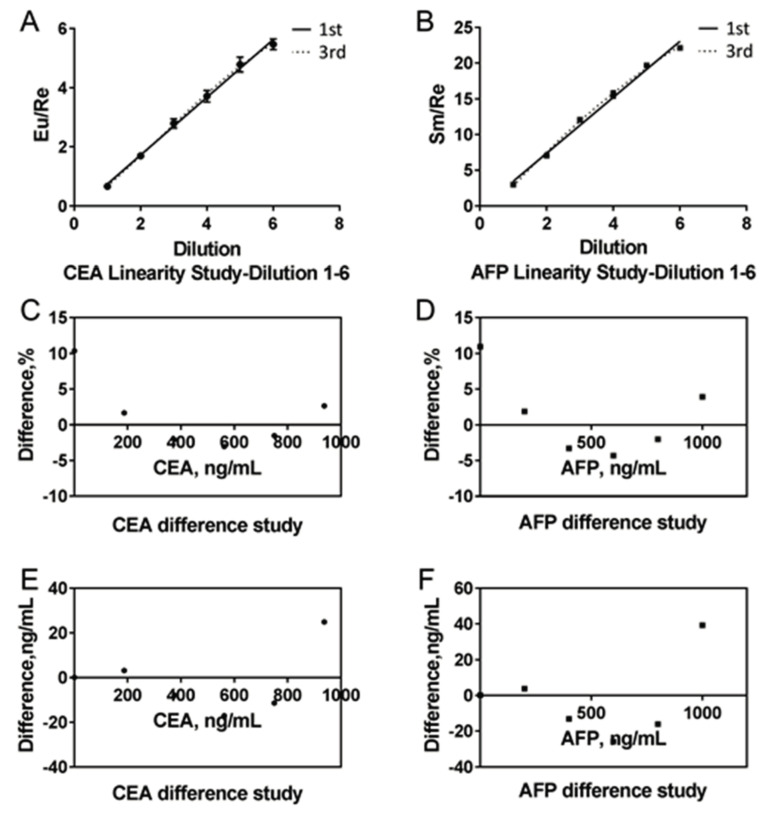
Evaluation of the linearity. (**A**) The first- and the third-order models of CEA, the equation of the two models was Y = 0.9761 X − 0.2292 and Y = −0.2239 + 0.818·X + 0.0948·X^2^ − 0.0121·X^3^, respectively; (**B**) The first- and the third-order models of AFP, the equation of the two models was Y = 4.055X − 0.764 and Y= −1.435 + 4.348·X + 0.0529·X^2^ − 0.0159·X^3^, respectively. (**C**,**D**) The differences between the first- and the third-order models for CEA and AFP were all less than 12.5%. (**E**,**F**)The concentration differences between the first- and the third-order models for CEA and AFP.

With regard to the linearity of the system, as is shown in Figure 4, the percentage deviations of all the dilutions for CEA and AFP were all less than 12.5%, which indicated good linearity from 2 ng/mL to 940 ng/mL for CEA, and from 1.5 ng/mL to 1000 ng/mL for AFP.

### 2.4. Evaluation of the Imprecision

**Figure 5:**
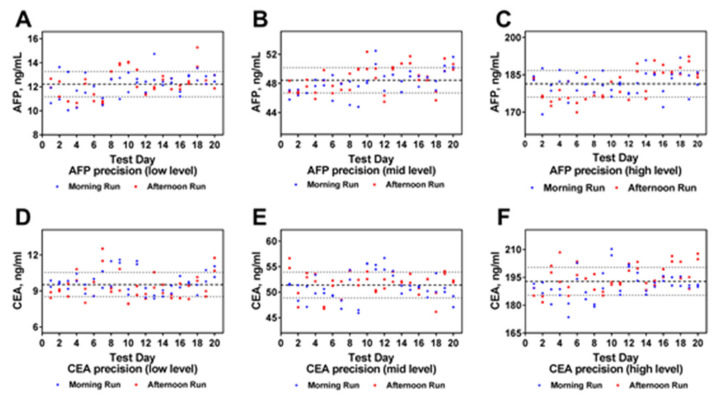
Evaluation of the assay imprecision. (**A**) The precision evaluation of CEA at low concentration; (**B**) The precision evaluation of CEA at middle concentration; (**C**) The precision evaluation of CEA at high concentration; (**D**) The precision evaluation of AFP at low concentration; (**E**) The precision evaluation of AFP at middle concentration; (**F**) The precision evaluation of AFP at high concentration.

Regarding the imprecision of the proposed method, the intra-assay (*n* = 80) and inter-assay (*n* = 40) CVs for CEA samples were 6.58% and 10.62% at low concentration (9.53 ng/mL), 2.51% and 4.97% at intermediate concentration (51.40 ng/mL), and 1.90% and 3.89% at high concentration (192.81 ng/mL), respectively; the intra-assay (*n* = 40) and inter-assay CVs (*n* = 80) for AFP samples were 6.32% and 8.69% at low concentration (12.22 ng/mL), 2.04% and 3.60% at intermediate concentration (48.44 ng/mL), and 2.01% and 2.95% at high concentration (181.38 ng/mL), respectively (Figure 5, Supplemental Tables S6 and S7).

### 2.5. Evaluation of the Accuracy

With regard to accuracy, all the detection results for the quality control serum from the NCCL and clinical samples were within the allowable range (Supplemental Figure S4). Regarding the specificity of the proposed assay, all the cross-reactivity rates for CEA and AFP were less than 0.1% (Supplemental Tables S8 and S9). The recovery rates of the immunoassay for CEA and AFP detection were between 92% and 107%, which indicated good recovery performance (Supplemental Table S10).

The interfering substances (including triglyceride, bilirubin and hemoglobin) at various concentrations had little influence on detection, and no bias greater than 10% was observed (Supplemental Figures S5 and S6). Regarding the stability, two levels of serum were measured daily, and nearly all the biases of CEA and AFP were less than 10%, which indicated that the assay had good stability (Supplemental Figure S7).

### 2.6. Establishment of Reference Interval

**Figure 6:**
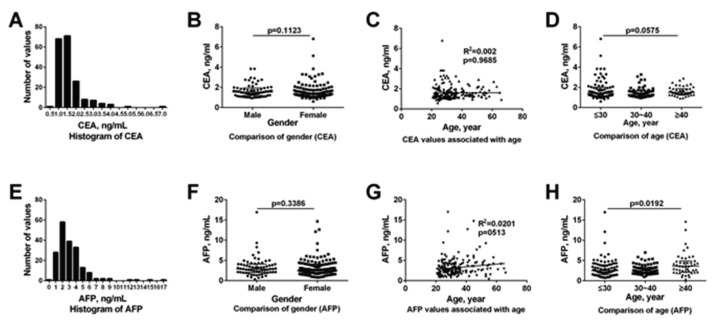
Reference interval of CEA and AFP. (**A**) Histogram of CEA values; (**B**) Comparison of gender for CEA, there was no significant difference between different gender group for CEA (*p* = 0.1123); (**C**) CEA values associate with age, CEA values were not correlated with age (R^2^ = 0.002; *p* = 0.9685); (**D**) Comparison of age for CEA, there was no significant difference between different age group for CEA (*p* = 0.575); (**E**) Histogram of AFP values; (**F**) Comparison of gender for AFP, there was no significant difference between different gender group for AFP (*p* = 0.3386); (**G**) AFP values associate with age, AFP values were not correlated with age (R^2^ = 0.0020; *p* = 0.0513); (**H**) Comparison of age for AFP, there was significant difference between different age group for AFP (*p* = 0.0192).

The detection results were firstly divided into different gender groups (males, females) and Figure 6 shows that there was no significant difference between males and females for CEA (*p* = 0.1123) and AFP (*p* = 0.3386). In addition, the Harris–Boyd test did not indicate partitioning between males and females. Since z < z* (Supplemental Table S11), the groups were not separated but combined (*n* = 190).

Regarding to different age groups, the Spearman’s rank correlation analysis indicated that there was no correlation between the values and age for both CEA (R^2^ = 0.002; *p* = 0.9685) and AFP (R^2^ = 0.0020; *p* = 0.0513). As for CEA, the Kruskal–Wallis test results indicated that there was no significance between different age groups, and the Harris–Boyd test results between different age groups did not demonstration partition. Thus, the subgroups were combined and the RI for CEA was 3.84 ng/mL. Regarding to AFP, there was significance between different age groups and the Harris–Boyd test results between different age groups demonstration partition. The subgroups were also combined and the RI for AFP was 9.94 ng/mL.

### 2.7. Detection of Clinical Samples

**Figure 7:**
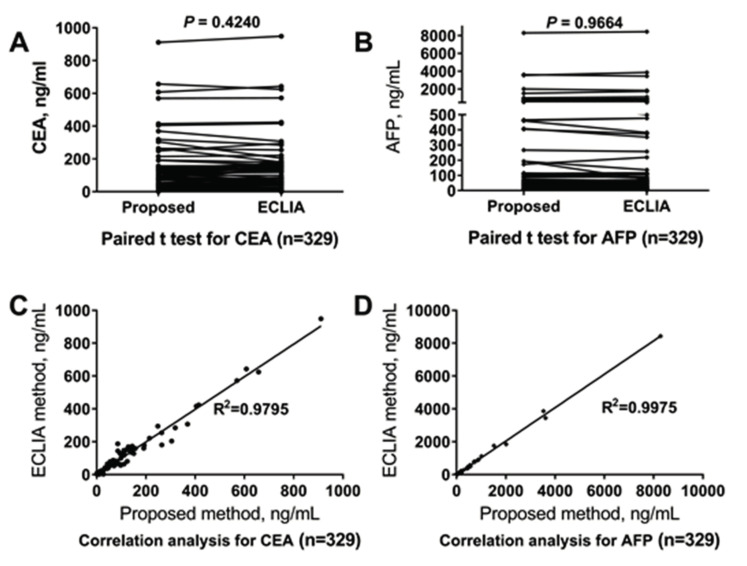
Clinical application of the immunoassay. (**A**) Paired t-test of the proposed method and the electrochemiluminescent immunoassay (ECLIA) method for CEA, the *p*-value was 0.4240; (**B**) Paired t-test of the proposed method and the ECLIA method for AFP, the *p*-value was 0.9664; (**C**) Correlation analysis of the proposed method and the ECLIA method for CEA, the correlation coefficient of them was 0.9795; (**D**) Correlation analysis of the proposed method and the ECLIA method for AFP, the correlation coefficient of them was 0.9975.

As shown in Figure 7, there was no significant difference between the proposed method and the ECLIA method for the analysis of CEA (*p* = 0.4240) and AFP (*p* = 0.9664). The correlation coefficient was 0.9795 for CEA and 0.9975 for AFP between our proposed method and the commercially available ECLIA method.

## 3. Discussion

Over the years, the development of immunoassays has gone through different stages. Although the initial RIA has the disadvantage of radioactive pollution, it also has certain advantages. For example, the volume of the element is negligible compared to the antibody, thus there is no steric hindrance during the reaction progress and the analysis speed is high. Fortunately, the stable isotope elements labeling strategy used in the ICP-MS-based immunoassay has inherited the advantage and avoided the disadvantages of RIA. Compared with other immunoassays (ECLIA, FIA, CLIA, etc.), the ICP-MS-based immunoassay has several unique advantages and the potential capability for multiplex analysis [22,23,34,35]. Although many studies have been reported for the ICP-MS-based immunoassay, these was no report of multiplex detection involve in standard evaluation and clinical sample detection with the guidance of CLSI documents [23]. Thus, CEA and AFP were selected to verify the clinical utility of the ICP-MS-based duplex immunoassay in this study.

For the application of the ICP-MS which was used as the detector of the multiplex immunoassay in clinical laboratories, there are two aspects of vital importance: (1) the reliability of the analyzer; (2) the overlap extent between different labels used for different antibodies. Thus, the accuracy and reliability of the ICP-MS analyzer was assessed first. According to the results in Supplemental Figure S2, Re^3+^ was selected as the internal control element. Assessment of the precision of the analyzer indicates that the CVs of different elements were all less than 5%. Similarly, with Eu, the carryover of Sm was less than 0.5%, which indicates that the ICP-MS instrument could be applied to clinical detection. Regarding to the overlap extent between different labels, as is shown in Figure 1, the relative signal value of Eu and Sm will not be affected by another element, regardless of the concentration of interference elements. The experiment results indicated that analysis of Sm and Eu do not suffer from mass spectral interferences. The most likely explanation is: (1) In the experiments, ^147^Sm isotope and ^153^Eu isotope were analyzed. Since masses of both isotopes are unique, isobaric interferences should not be considered; (2) The probability for the present of polyatomic interferences is fairly low, because both the concentration of reactant ions (such as Cd, Ag, Xe, Te, Ba, In, etc) in the system (most of them have been removed during immunoassay), and the yield of reaction to produce those polyatomic ions are extremely low; (3) The resolution of instrument used in the experiments was around 0.3–0.7 atomic mass units, which is good enough to remove spectral interferences resulted from overlapping of adjacent isotopes, for instance, ^152^Sm (mass: 151.9197) and ^154^Sm (mass: 153.9222) on ^153^Eu (mass: 152.9212); (4) The detection process of the two elements is performed separately in the ICP-MS analyzer, so there will be no competition between them. From the discussion above, the contribution of spectral interferences during analysis of Sm and Eu using ICP-MS to deviation of experiment results should be ignored.

The present study indicated that clinical application of the proposed ICP-MS-based duplex immunoassay is acceptable from both analytical and clinical perspectives for the following reasons: (a) The LoD, LLoQ, ULoQ, AMR, CRR of the immunoassay were 0.93 ng/mL, 2 ng/mL, 940 ng/mL, 2–940 ng/mL, 2–9400 ng/mL for CEA, and 0.34 ng/mL, 1.5 ng/mL, 1000 ng/mL, 1.5–1000 ng/mL, 1.5–10,000 ng/mL for AFP, respectively, which was sufficient for analysis of almost all the clinical samples. (b) The percentage deviation of all the dilutions for CEA and AFP was less than 12.5% during linearity evaluation (Figure 4). (c) Inter- and intra-assay variations were in accordance with clinical laboratory requirements for both CEA and AFP (Figure 5 and Supplemental Table S7), which means that clinically significant error of the proposed duplex assay is small. (d) Regarding to accuracy evaluation, biases for detection of quality control serum from the NCCL and clinical samples for both CEA and AFP were all within the allowable range (Supplemental Figure S4). Together with the results of the recovery rate evaluation study, the results of accuracy estimate indicated that the proposed duplex assay is a reliable method of high accuracy. (e) As is shown in Supplemental Tables S8 and S9, Figures S5 and S6, cross-reactivity and interference evaluation revealed that the proposed assay will not be affected when the samples were incubated with other tumor markers and substances. (f) The total time needed (including 7 min for centrifugation, 30 min for reaction, nearly 5 min for washing, and 1 min for element analysis) for the proposed assay was less than 45 min, which is faster than almost all the manual immunoassays (such as ELISA). (g) Regarding to clinical sample detection, there was no significant difference of the results between the proposed duplex assay and the ECLIA methods for both CEA and AFP. At the same time, the correlation coefficient of the results for both CEA and AFP between the two methods were all more than 0.97 (Figure 7), which indicated that the proposed assay could be used for the clinical determination of CEA and AFP level in human serum.

With regard to the establishment of RIs for CEA and AFP, the measured values were first divided into male and female groups, the Harris–Boyd test did not indicate partitioning, and there was no significant difference of CEA and AFP values between different gender groups. After that, CEA and AFP values were divided into different age groups. The Harris–Boyd test did not indicate partitioning for CEA. The RI of CEA in this study, which is determined using the 97.5th percentiles based on 190 subjects according to the Shapiro–Wilk test, is 0–3.84 ng/mL. Regarding to AFP, the Harris–Boyd test indicated partitioning for different age groups. However, although there was a significant difference of AFP values between different age groups from a statistical point of view, there was no obvious clinical significance of the difference from a medical point of view. At the same time, the sample size of different age group is not sufficient. Thus, the RI of AFP was defined as 0–9.94 ng/mL based on a total of 190 subjects according to the Shapiro–Wilk test. The RI of CEA and AFP claimed by the manufacturer is 0–5 ng/mL and 0–20 ng/mL, respectively, which is different from the results of our study [36]. There are two reasons for the difference. First, the population used in the calculation for RIs of the ECLIA kit was mainly from Europe or America, rather than China. The levels of biomarkers secreted in the body may vary depending on the physique in different areas. Second, the sample size of our study is different from that of the ECLIA kits.

There were also some limitations to this study. First, the clinical samples were collected in our hospital, and multi-center testing was not performed, which may make our study less representative. Second, the sample size of the study in the establishment of RIs was not large enough to keep all the subgroups has more than 120 samples. Third, the ICP-MS analyzer used was only from one company, which cannot reflect the reliability of all the instruments.

## 4. Conclusions

In this study, based on our previous study of ICP-MS-based simplex immunoassay, we have further successfully established a duplex detection system. Evaluation of the ICP-MS-based duplex immunoassay demonstrated its performance characteristics (including good specificity, high sensitivity, good stability, wide detection range, and high accuracy), which undoubtedly proved the clinical application of the proposed duplex immunoassay. This study further lays the foundation for the clinical application of the ICP-MS-based immunoassay, and promotes the combination of mass spectrometry technology and immunoassay technology. More importantly, this study provides a new strategy for simultaneous multi-index detection in clinical laboratories.

## 5. Materials and Methods

### 5.1. Instruments and Reagents

The iCAP Q ICP-MS instrument was supplied by Thermo Fisher Scientific Co., Ltd., Beijing, China. The magnetic frame was bought from Thermo Fisher Scientific Co., Ltd., Beijing, China. The Orbital Shaker Incubator was purchased from Suzhou Peiying Experimental Equipment Co., Ltd., Suzhou, China. The 1.5 mL and 0.6 mL Ep tubes were bought from Axygen Biotechnology Co., Ltd., Beijing, China. The rare earth elements (REEs): Eu, Sm, Tb, Ho, In, Rh and Re standard solutions (1000 µg/mL) were purchased from the National Research Center for Certified Reference Material, Beijing, China. Streptavidin functional magnetic beads were purchased from the Roche Co., Ltd., Basel, Switzerland. The CEA antibodies were purchased from Fapon Biotech Inc. Co., Ltd., Shenzhen, China and labeled by Jiangsu Institute of Nuclear Medicine, Jiangsu, China. The AFP antibodies were purchased from and labeled by the Jiangsu Institute of Nuclear Medicine, Jiangsu, China. The PBS buffer and washing buffer were purchased from the Jiangsu Institute of Nuclear Medicine, Jiangsu, China. The triglyceride, bilirubin and hemoglobin reagents were purchased from Solarbio Biotechnology Co., Ltd., Beijing, China. The standard quality control serum was purchased from the National Center for Clinical laboratories (NCCL) in China. All chemicals and reagents used were at least of analytical and or higher grade.

### 5.2. Clinical Samples

A total of 329 clinical samples were collected from the PLA General Hospital in China from December, 2018 to December 2019. Among that, 164 samples were from males and 165 samples were from females, detailed information is shown in Supplemental Table S1. In addition, a total of 190 blood samples (serum) from healthy individuals (65 males, 125 females, aged 22–65 years) were used to establish the RIs for CEA and AFP of the proposed assay. The study was approved by the Ethical Committee of PLA General Hospital (number S2018-282-02).

### 5.3. Evaluation of the ICP-MS Analyzer

Eu^3+^ and Sm^3+^ were selected as the potential labeling element for CEA and AFP, respectively. The operation parameters of the ICP-MS analyzer are shown in Table 1. In order to eliminate the impact of some interference situations (such as salts and incomplete injection) on the test results, Rh^3+^, In^3+^, Tb^3+^, Ho^3+^ and Re^3+^ were selected as potential quality control elements according to previous reports [34]. In addition, the Eu-labeled CEA antibody and the Sm-labeled AFP antibody were tested 50 times to evaluate the imprecision (presented as relative standard deviation/coefficient of variation (CV)) of the analyzer. In order to show that the detection signals of the instrument were from the labeled antibodies, the Eu and Sm signal values of different substances involved in the reaction were detected by ICP-MS analyzer, including serum, PBS buffer, water, and antibodies. In addition, the linearity of the two elements was evaluated with a high concentration of another element to reflect the overlap of different elements. Similarly, with Eu^3+^, carryover of the analyzer for Sm3+ was evaluated according to the CLSI document H26-A2 [37].

### 5.4. Assay Protocol and Its Optimization

**Figure 8:**
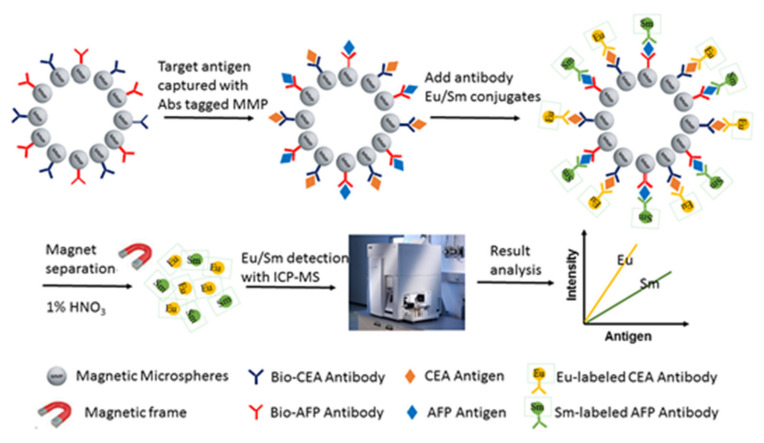
The principle of the ICP-MS-based duplex immunoassay. The immunoassay reaction was carried out in a tube and MMPs were used as the carrier. Firstly, the biotinylated antibodies of CEA and AFP were immobilized on the MMPs to form immunomagnetic beads (IMBs) via biotin and streptavidin reactions, respectively. Then the IMBs for CEA and AFP were mixed and 30 microL of the mixed IMBs was added to a tube. After that, 30 microL of serum was added to the tube for the reaction. After washing, the Eu^3+^-labeled CEA antibody and Sm^3+^-labeled AFP antibody were added to the tube simultaneously. Reactions of the different steps were carried out at 37 °C. The IMBs-antigen-element-labeled antibody complexes for CEA and AFP were retained by the adsorption force of the magnetic frame and the unresponsive antibodies were washed off. Then, 1% HNO_3_ (*v*/*v*) was added to the tube to release the element from the antibody, and the concentration of the element was detected by ICP-MS analysis. The antigen content for CEA and AFP were proportional to signal intensities of Eu and Sm, respectively.

As shown in Figure 8, the principle of the established system was a sandwich immunoassay. For optimization, based on our previous experiment, two steps (the Eu^3+^-labeled CEA antibody and Sm^3+^-labeled AFP antibody were added after the antigen and bio-antibody binding reaction step was finished) of the detection process were selected [31]. The ratio of the captured antibody/MMP for CEA and AFP were optimized firstly. Then, the amount of immunomagnetic beads (IMBs) for CEA and AFP were optimized using CEA and AFP standard sample (a series of concentrations), excess Eu^3+^-labeled anti-CEA antibody and excess Sm^3+^-labeled anti-AFP antibody. In addition, the amount of Eu^3+^-labeled anti-CEA antibody and Sm^3+^-labeled anti-AFP antibody were optimized using a CEA and AFP standard sample (500 ng/mL for CEA and 500 ng/mL for AFP), and excess IMBs of CEA and AFP. The reaction time (including 5 min, 10 min, 15 min, 20 min, 30 min, 40 min and 50 min) and PBS buffer volume (including 30 microL, 60 microL, 90 microL, 120 microL, 150 microL and 180 microL, 210 microL, 240 microL) of the sample reaction step and labeled antibody reaction step were also optimized, respectively.

### 5.5. Evaluation of the Calibration Curves

Calibration curves of CEA and AFP were established by the detection of a series of standard products (5 ng/mL, 10 ng/mL, 20 ng/mL, 30 ng/mL, 40 ng/mL, 50 ng/mL, 100 ng/mL, 200 ng/mL, 400 ng/mL, 600 ng/mL and 800 ng/mL). As for the limit of detection (LoD), blank samples without CEA and AFP antigen were measured 60 times at different conditions, and the LoD of CEA and AFP was defined as the concentration corresponding to a signal three SD above the mean of the blank signal. According to CLSI document EP17-A2, the lower limit of quantification (LLoQ) was defined as the lowest concentration at which the imprecision (CV) was < 20% (LLoQ), with the allowable bias less than 8.33% [38]. According to CLSI document EP34-A, a clinical sample with a high concentration of CEA and AFP (nearly 600 ng/mL) was diluted for different ratios (2, 5, 10, 15, 20 and 30 times) to determine the allowable dilution ratio, and the dilution ratio was acceptable when the biases of CEA and AFP detection did not exceed 8.38% simultaneously [39]. Based on that, the upper limit of the clinical reportable range (CRR) for CEA and AFP was defined as their ULoQ multiplied by the acceptable dilution ratio.

According to CLSI document EP06-A2, linearity was evaluated for six pools of CEA and AFP serums: serum at two levels, the LLoQ concentration and ULoQ concentration, and four pools from the mixture of the mentioned serum with the ratio of 4:1, 3:2, 2:3 and 1:4. Linear-fit values of CEA and AFP were calculated using the equation of the best fitted line, and percentage deviation for CEA and AFP from the linearity of each pool was calculated using the equation: 100% − (predicted value/linear fit value) × 100% [40].

### 5.6. Evaluation of the Imprecision

Regarding the imprecision of the assay, three concentrations of samples (low, intermediate and high level) were selected. According to CLSI document EP05-A3, two runs of one plate each were performed daily for nonconsecutive 20 working days, and the samples on each plate were measured in duplicate [41].

### 5.7. Evaluation of the Accuracy

According to CLSI document EP09-A3, the accuracy for CEA and AFP detection of the proposed immunoassay was evaluated by detecting standard quality control serums at five levels and 20 clinical samples at various concentrations [42]. The specificity of the system for CEA and AFP detection was evaluated by the cross-reactivity through measuring other possible interfering tumor markers (CEA, AFP, carbohydrate antigen 12-5 (CA 12-5), and carbohydrate antigen 19-9 (CA 19-9)) [8]. The recovery rates (RR) of the proposed assay for CEA and AFP were assessed by clinical samples at different concentrations and evaluated by the formula: RR = (Predicated value − original value)/Added value × 100%.

The interference experiment was carried out reference to CLSI guideline EP7-A2 with different interfering substances (including triglyceride, bilirubin and hemoglobin) for six pools or eight pools mixed by the blank and high concentrations [43]. The high concentrations of triglyceride, bilirubin and hemoglobin were 17.3 mmol/L, 342 μmol/L and 4 g/L, respectively. As for the stability, the reagents were stored at 37 °C for 7 days (equal to 4 °C for 1 year) and used to test three levels of serums every day to estimate the bias of each result.

### 5.8. Establishment of Reference Interval

According to CLSI document C28-A2, reference intervals of CEA and AFP of the proposed assay were established by testing serum samples from 190 healthy test subjects (65 males, 125 females, aged 26–66 years) [44]. The D/R ratio was used to estimate whether all the data should be kept (D/R < 1/3), where D was the difference between an extreme value (large or small) and the next largest (or smallest) value, and R was the range of all observations. If not, the extreme value should be deleted and the data estimated again. The samples were firstly divided into different gender and age groups. Then according to Harris–Boyd method, the z values with the standard deviation and a calculated z for two groups were used to determine whether each group is sufficient different statistically to its own grouping. If the results of the Harris–Boyd test did not demonstration partition, the subgroup should be combined. The value of RIs for CEA and AFP were set as the 97.5% percentile.

### 5.9. Clinical Sample Detection

A total of 329 clinical samples were analyzed by the proposed assay, and the CEA and AFP results were compared with those of the ECLIA.

### 5.10. Statistical Analysis

All analyses were performed using SPSS 17.0 (SPSS, Chicago, IL, USA) and GraphPad Prism software (GraphPad, San Diego, CA, USA). The Shapiro–Wilk normality test was used to evaluate whether the data were normally distributed. The nested analysis of variance (ANOVA) was used to calculate intra- and inter-assay components of imprecision. Analysis of the differences between groups was carried out using unpaired t-test, paired t-test, one-way ANOVA, or Mann–Whitney test, as appropriate. The correlation between age and serum CEA/AFP was assessed by Spearman’s rank correlation coefficient for the establishment of RI. The paired t test and Pearson’s correlation coefficient were used to evaluate the difference and associations respectively between the proposed method and the ECLIA method. Values of *p* < 0.05 were defined as statistically significant.

## Figures and Tables

**Table 1:** ICP-MS operation parameters.

Parameter	Value
cool gas flow (L/min)	13
auxiliary gas flow (L/min)	0.7
nebulizer gas flow (L/min)	0.96
sample uptake (s)	38
dwell time (ms)	0.05
channel	3
number of repeats per sample	3
PC detector voltage (V)	1265
RF power (W)	1578.61
analogue detector voltage (V)	−1960

## Supplementary Materials

Figure S1: Selection of quality control element, Figure S2: Analysis of the source of Eu/Sm signals, Figure S3: Determination of ULoQ for CEA and AFP, Figure S4: Evaluation of the accuracy of the assay, Figure S5: Interference testing of the assay for CEA, Figure S6: Interference testing of the assay for AFP, Figure S7: Stability of the immunoassay, Table S1: Information of clinical samples, Table S2: Carryover of ICP-MS instrument for Sm detection, Table S3: Determination of the LLoQ (n = 20), Table S4: The allowable dilution ratio of CEA (n = 3), Table S5: The allowable dilution ratio of AFP (n = 3), Table S6: Intra-assay and inter-assay imprecision analysis for CEA, Table S7: Intra-assay and inter-assay imprecision analysis for AFP, Table S8: Specificity of the immunoassay for CEA (n = 3), Table S9: Specificity of the immunoassay for AFP (n = 3), Table S10: Recovery rate of the immunoassay (n = 3), Table S11: Harris-Boyd’s test for different subgroups.
