# Supplementary material for: Evaluation of an Element-Tagged Duplex Immunoassay Coupled with Inductively Coupled Plasma Mass Spectrometry Detection: A Further Study for the Application of the New Assay in Clinical Laboratory
Source: Molecules. 2020 Nov 17;25(22):5370. doi: 10.3390/molecules25225370 (PMC7698432; doi:10.3390/molecules25225370)
Supplement: Supplementary file 1 [file molecules-25-05370-s001.pdf]

# Supporting Information

**Title:** Evaluation of an Element-Tagged duplex immunoassay Coupled with Inductively Coupled Plasma Mass Spectrometry detection: A further study for the application of the new assay in clinical laboratory.

**Keywords:** ICP-MS based duplex immunoassay; CEA; AFP; evaluation; performance; clinical sample detection

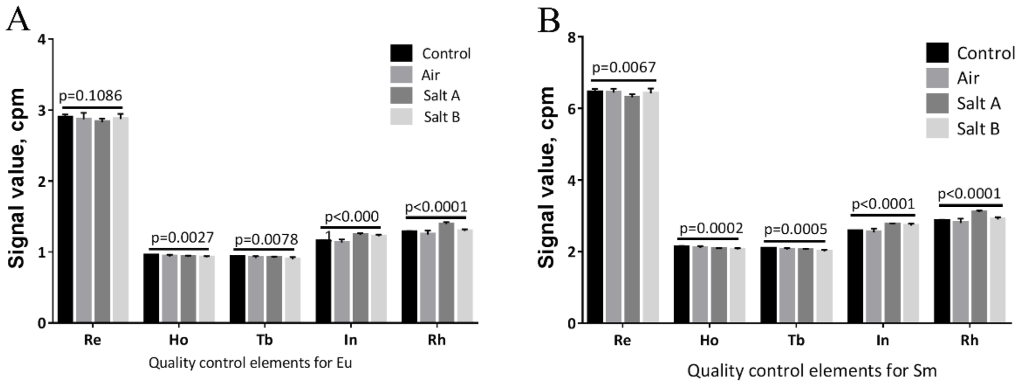


Figure S1 Selection of quality control element

Note: The air, salt A and salt B conditions shown in the figure refer to incomplete injection, low concentration of salty interfering substances (the sample contains 10% of 1 ×PBS) and high concentration of salty interfering substances (the sample contains 10% of 25 ×PBS), respectively. There was no significant difference between the four conditions for Eu when the data was corrected by the element Re (p=0.1086), although there was significant difference between the four conditions for Sm. Re can be the quality control element compare to the other four elements Ho, Tb, In and Rh.

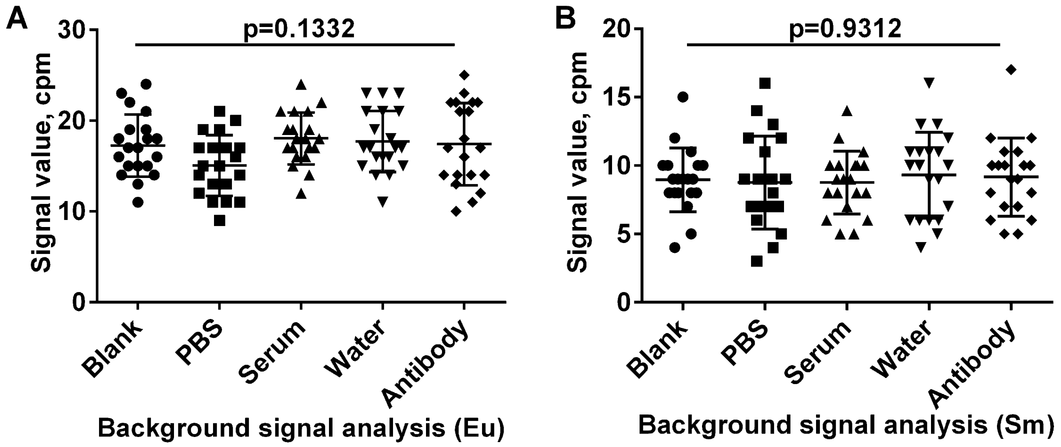


Figure S2 Analysis of the source of Eu/Sm signals

Note: A) Background signal analysis for Eu, there’s no significant difference between the signal of blank and signals from PBS, serum, water and antibody; B) ackground signal analysis for Sm, there’s no significant difference between the signal of blank and signals from PBS, serum, water and antibody.

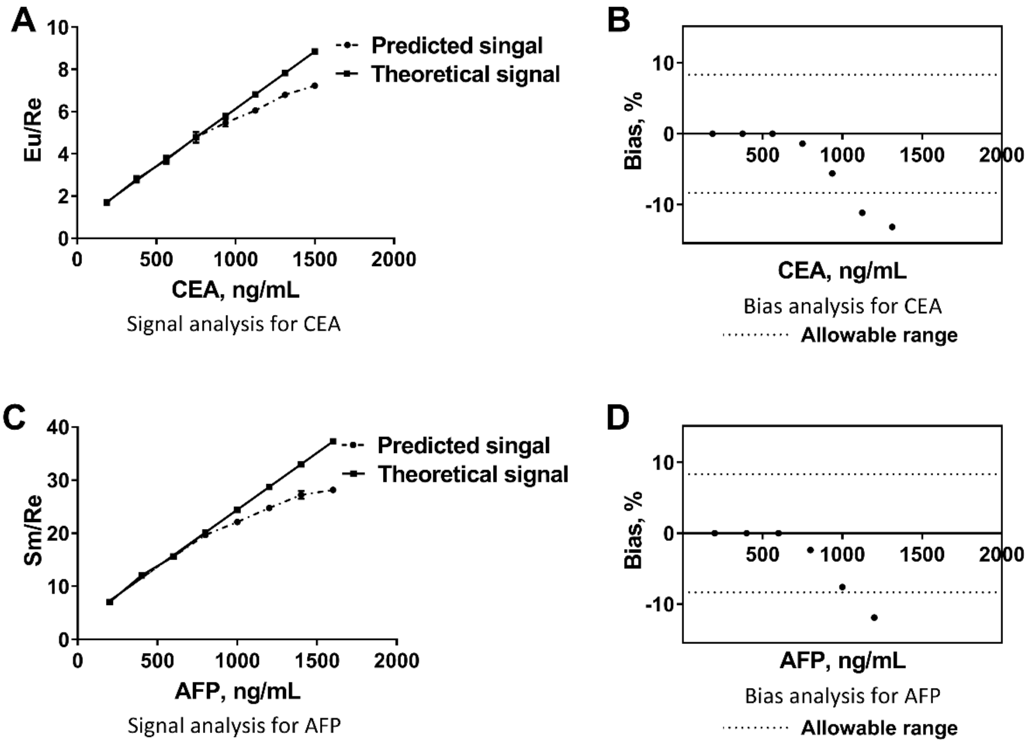


Figure S3 Determination of ULoQ for CEA and AFP

Note: Bias = (Predicted value - actual concentration)/ actual concentration × 100%. (A) Signal analysis for CEA, along with the increase of CEA concentration, the difference between the predicted signal value and the theoretical signal value increased as well; (B) Bias analysis for CEA, along with the increase of CEA concentration, the bias increased as well and the bias was blew the allowable range when the concentration of CEA was 900 ng/mL; (C) Signal analysis for AFP, along with the increase of AFP concentration, the difference between the predicted signal value and the theoretical signal value increased as well; (D) Bias analysis for AFP, along with the increase of AFP concentration, the bias increased as well and the bias was blew the allowable range when the concentration of CEA was 1000 ng/mL.

Reference: NCCLS. Evaluation of the Linearity of Quantitative Measurement Procedures; A Statistical Approach; Approved Guideline. NCCLS document EP6-A. Wayne, PA: Clinical and Laboratory Standards Institute; 2003.

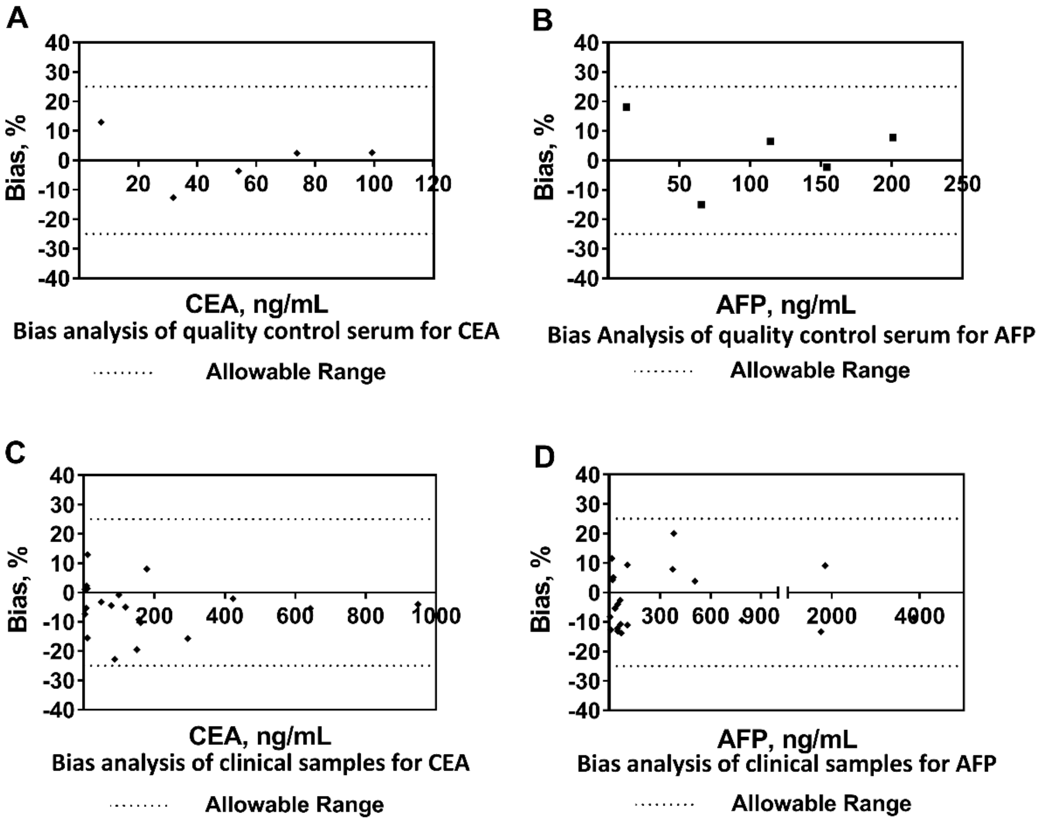


Figure S4. Evaluation of the accuracy of the assay.

Note: (A) Bias analysis of quality control serum for CEA, all the biases of the quality control serum did not exceed the allowable bias range; (B) Bias analysis of quality control serum for AFP, all the biases of the quality control serum did not exceed the allowable bias range; (C) Bias analysis of clinical samples for CEA, all the biases of the clinical samples did not exceed the allowable bias range; (D) Bias analysis of clinical samples for AFP, all the biases of the clinical samples did not exceed the allowable bias range.

Reference: CLSI. Measurement Procedure Comparision and Bias Estimation Using Patiengt Samples;Approved Guideline-Third Edition. CLSI document EP09-A3. Wayne, PA: Clinical and Laboratory Standards Institute; 2013.

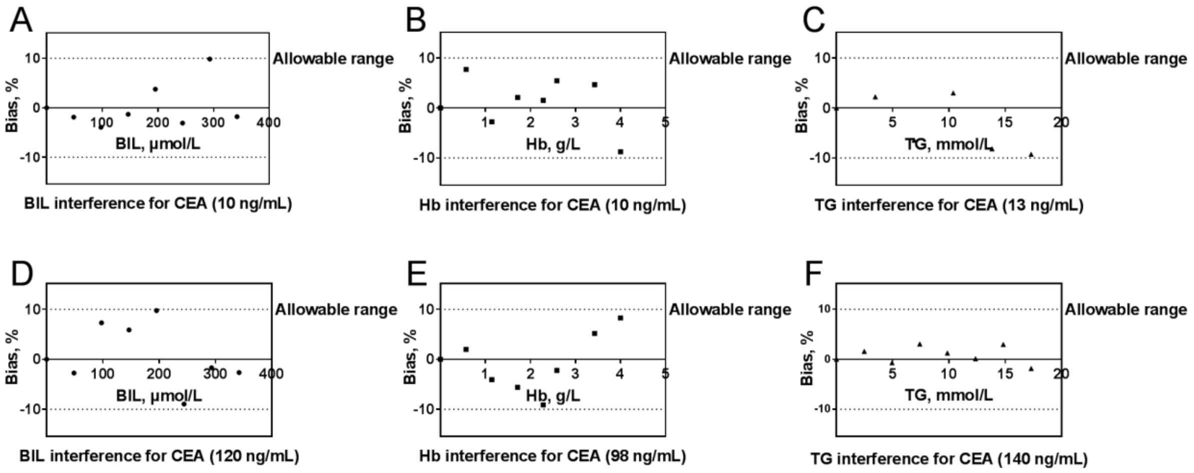


Figure S5 Interference testing of the assay for CEA

Note: (A) Bilirubin (BIL) interference for CEA (low level), biases of different concentrations of bilirubin were all within the allowable range; (B) Hemoglobin (Hb) interference for CEA (low level), biases of different concentrations of hemoglobin were all within the allowable range; (C) Triglyceride (TG) interference for CEA (low level), biases of different concentrations of triglyceride were all within the allowable range; (D) Bilirubin (BIL) interference for CEA (high level), biases of different concentrations of bilirubin were all within the allowable range; (E) Hemoglobin (Hb) interference for CEA (high level), biases of different concentrations of hemoglobin were all within the allowable range; (F) Triglyceride (TG) interference for CEA (low level), biases of different concentrations of triglyceride were all within the allowable range.

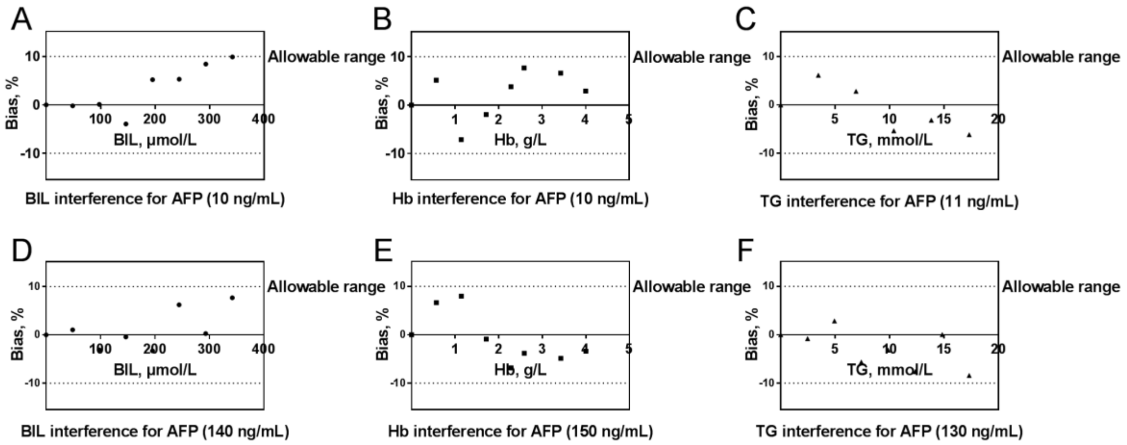


Figure S6 Interference testing of the assay for AFP

Note: (A) Bilirubin (BIL) interference for AFP (low level), biases of different concentrations of bilirubin were all within the allowable range; (B) Hemoglobin (Hb) interference for AFP (low level), biases of different concentrations of hemoglobin were all within the allowable range; (C) Triglyceride (TG) interference for AFP (low level), biases of different concentrations of triglyceride were all within the allowable range; (D) Bilirubin (BIL) interference for AFP (high level), biases of different concentrations of bilirubin were all within the allowable range; (E) Hemoglobin (Hb) interference for AFP (high level), biases of different concentrations of hemoglobin were all within the allowable range; (F) Triglyceride (TG) interference for AFP (low level), biases of different concentrations of triglyceride were all within the allowable range.

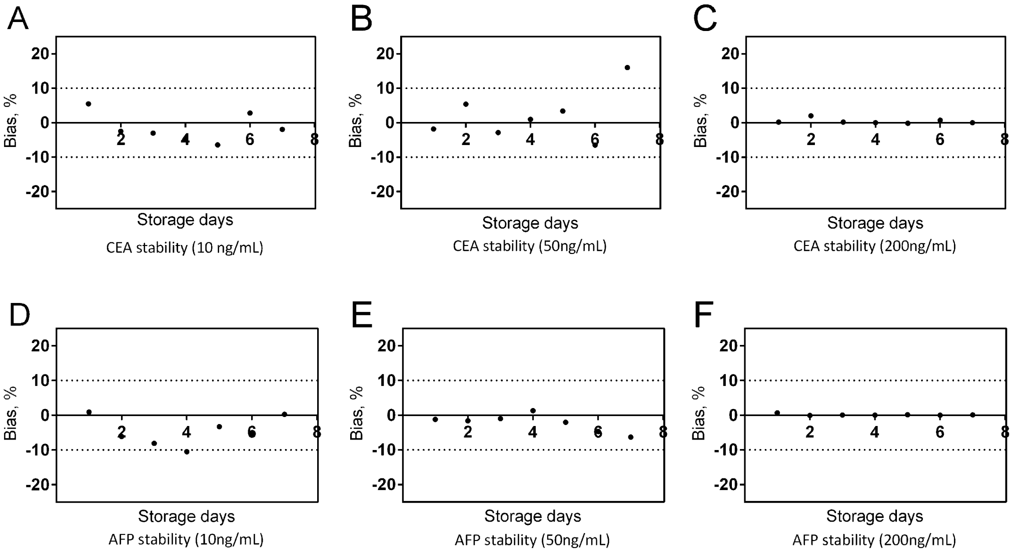


Figure S7 Stability of the immunoassay

Note: (A) CEA stability of low concentration, all the biases were less than 10%; (B) CEA stability of intermediate concentration, all the biases except the 7th day were less than 10%; (C) CEA stability of high concentration, all the biases were less than 10%; (D) AFP stability of low concentration, all the biases except the 4th day were less than 10%; (E) AFP stability of intermediate concentration, all the biases were less than 10%; (F) AFP stability of high concentration, all the biases were less than 10%.

Table S1 Information of clinical samples

| Groups | Sample number | CEA, ng/mL | | AFP, ng/mL | |
|---|---|---|---|---|---|
| | | Mean±SD | Range | Mean±SD | Range |
| All | 329 | 30.60±95.90 | 0.21~949.00 | 98.93±573.70 | 0.61~8428.00 |
| Male | | | | | |
| ≤30 | 29 | 2.22±1.97 | 0.59~11.38 | 10.00±40.37 | 0.84~219.80 |
| 31~50 | 52 | 17.42±46.12 | 0.49~221.20 | 42.63±111.90 | 0.69~506.70 |
| 51~70 | 67 | 46.96±80.82 | 0.52~423.70 | 342.30±1206.00 | 0.80~8428.00 |
| >70 | 16 | 118.5±170.40 | 0.21~643.00 | 229.90±524.90 | 1.13~1846.0 |
| Female | | | | | |
| ≤30 | 54 | 1.08±0.43 | 0.38~2.40 | 2.35±1.40 | 0.69~7.41 |
| 31~50 | 69 | 16.09±76.83 | 0.34~571.80 | 13.04±64.05 | 0.61~525.80 |
| 51~70 | 37 | 50.48±122.00 | 0.26~624.30 | 49.65±157.40 | 0.83~895.00 |
| >70 | 5 | 198.60±419.80 | 2.26~949.00 | 112.40±205.70 | 1.22~476.70 |

Table S2 Carryover of ICP-MS instrument for Sm detection

| Concentration differential times | *HTV*1 | *HTV*2 | *HTV*3 | *LTV*1 | *LTV*2 | *LTV*3 | Carryover, % |
|---|---|---|---|---|---|---|---|
| 10 | 30623.24 | 29723.17 | 29721.89 | 3160.20 | 3038.18 | 3030.72 | 0.49% |
| 10 | 3160.20 | 3028.18 | 3010.72 | 334.52 | 328.24 | 324.17 | 0.39% |
| 100 | 106278.07 | 105896.48 | 105574.28 | 1105.18 | 1097.56 | 1056.19 | 0.05% |
| 100 | 201961.37 | 201736.45 | 201656.97 | 2252.37 | 2220.17 | 2188.19 | 0.03% |

Note: The samples elevated "high target value"(HTV) concentration were processed in triplicate (*HTV* 1, *HTV*2, *HTV* 3 means the first, second and third value of the 3 serial detection, respectively), followed by three aspirations of "low target value" (LTV) samples (*LTV* 1, *LTV* 2, *LTV* 3 means the first, second and third value of the 3 serial detection, respectively). The carryover of the instrument was calculated as the formular: Carryover (%) = (*LTV*1-*LTV*3)/ (*HTV*3-*LTV*3) ×100%.

Reference: CLSI. Validation, Verification, and Quality Assurance of Automated Hematology Analyzers; Approved Standard-Second Edition. CLSI document H26-A2. Wayne, PA: Clinical and Laboratory Standards Institute; 2010.

Table S3 Determination of the LLoQ (n=20)

| Actual value, ng/ml | CEA | | | | | . | AFP | | | | |
|---|---|---|---|---|---|---|---|---|---|---|---|
| | 0.5 | 1.0 | 1.5 | 2.0 | 3.0 | | 0.5 | 1.0 | 1.5 | 2.0 | 3.0 |
| Predicted value, ng/ml | 0.63 | 1.17 | 1.69 | 1.89 | 3.07 | | 0.61 | 0.86 | 1.39 | 2.11 | 2.93 |
| SD | 0.203 | 0.263 | 0.293 | 0.211 | 0.313 | | 0.166 | 0.168 | 0.169 | 0.225 | 0.245 |
| CV, % | 32.27 | 22.47 | 17.32 | 11.36 | 9.87 | | 28.17 | 19.49 | 12.18 | 10.39 | 8.37 |
| Bias, % | 26.00 | 17.00 | 12.67 | 7.00 | 5.67 | | 22.00 | 14.00 | 7.33 | 5.50 | 2.33 |

Note: The imprecision of CEA at the concentration of 0.5 ng/mL, 1 ng/ mL, 1.5 ng/mL, 2 ng/mL and 3 ng/mL was 32.27%, 22.47%, 17.32%, 11.36% and 9.87%, respectively; the imprecision of AFP at the concentration of 0.5 ng/mL, 1 ng/mL, 1.5 ng/mL, 2 ng/mL and 3 ng/mL was 28.17%, 19.49%, 12.18%, 10.39% and 8.37%. Thus, the LLoQ of the assay was 2 ng/mL for CEA and 1.5 ng/mL for AFP.

Reference: CLSI. Evaluation of Detection Capability for Clinical Laboratory Measurement Procedures; Approved Guideline-Second Edition. CLSI document EP17-A2. Wayne, PA: Clinical and Laboratory Standards Institute; 2012.

Table S4 The allowable dilution ratio of CEA (n=3)

| dilution ratio | **1** | **2** | **5** | **10** | **15** | **20** | **30** |
|---|---|---|---|---|---|---|---|
| Predicted, ng/ml | 617.14 ±14.58 | 626.62± 28.87 | 646.04 ±9.08 | 656.36 ±8.78 | 674.58 ±17.15 | 680.91 ±21.26 | 696.80 ±6.30 |
| Difference, ng/ml | 0.00 | 9.48 | 28.90 | 39.22 | 57.44 | 63.77 | 79.66 |
| Bias, % | 0.00 | 1.54 | 4.68 | 6.35 | 9.31 | 10.33 | 12.91 |

Note: Bias= (Detective value- actual concentration)/ actual concentration×100%. Along with the increase of the dilution ratio, the bias of the proposed method increased gradually.

Reference: CLSI. Establishing and Verifying an Extended Measuring Interval Through Specimen Dilution and Spiking, 1st Edition. CLSI document EP34. Wayne, PA: Clinical and Laboratory Standards Institute; 2018.

Table S5 The allowable dilution ratio of AFP (n=3)

| dilution ratio | **1** | **2** | **5** | **10** | **15** | **20** | **30** |
|---|---|---|---|---|---|---|---|
| Predicted, ng/ml | 605.88 ±6.55 | 633.00 ±9.54 | 643.76 ±13.24 | 646.96 ±19.63 | 669.31 ±23.57 | 711.45 ±32.60 | 705.14 ±34.11 |
| Difference, ng/ml | 0.00 | 27.11 | 37.88 | 41.08 | 63.43 | 105.57 | 99.26 |
| Bias, % | 0.00 | 4.48 | 6.25 | 6.78 | 10.47 | 17.42 | 16.38 |

Note: Bias= (Detective value- actual concentration)/ actual concentration×100%. Along with the increase of the dilution ratio, the bias of the proposed method increased gradually.

Reference: CLSI. Establishing and Verifying an Extended Measuring Interval Through Specimen Dilution and Spiking, 1st Edition. CLSI document EP34. Wayne, PA: Clinical and Laboratory Standards Institute; 2018.

Table S6 Intra-assay and inter-assay imprecision analysis for CEA

| Sample | Mean, ng/mL | Intra-assay (n=80) | | Inter-assay (n=40) | |
|---|---|---|---|---|---|
| | | SD, ng/mL | CV, % | SD, ng/mL | CV, % |
| Low concentration | 9.53 | 0.63 | 6.58 | 1.01 | 10.62 |
| Mid concentration | 51.40 | 1.29 | 2.51 | 2.56 | 4.97 |
| High concentration | 192.81 | 3.66 | 1.9 | 7.50 | 3.89 |

Reference: CLSI. Evaluation of Precision of Quantitative Measurement Procedures; Approved Guideline-Third Edition. CLSI document EP05-A3. Wayne, PA: Clinical and Laboratory Standards Institute; 2014.

Table S7 Intra-assay and inter-assay imprecision analysis for AFP

| Sample | Mean, ng/mL | Intra-assay (n=80) | | Inter-assay (n=40) | |
|---|---|---|---|---|---|
| | | SD, ng/mL | CV, % | SD, ng/mL | CV, % |
| Low concentration | 12.22 | 0.77 | 6.32 | 1.06 | 8.69 |
| Mid concentration | 48.44 | 0.99 | 2.04 | 1.74 | 3.60 |
| High concentration | 181.38 | 3.65 | 2.01 | 5.34 | 2.95 |

Reference: CLSI. Evaluation of Precision of Quantitative Measurement Procedures; Approved Guideline-Third Edition. CLSI document EP05-A3. Wayne, PA: Clinical and Laboratory Standards Institute; 2014.

Table S8 Specificity of the immunoassay for CEA (n=3)

| Compound | Concentration, ng/ml | Predicted values, ng/ml | Cross-reactivity, % |
|---|---|---|---|
| AFP | 2600 | 1.92 ± 0.27 | 0.074 |
| CA19-9 | 1480 | 1.16 ± 0.31 | 0.078 |
| CA125 | 1200 | 0.98 ± 0.29 | 0.082 |

Note: The cross-reactivity rates of different compounds were all less than 0.10%.

Table S9 Specificity of the immunoassay for AFP (n=3)

| Compound | Concentration, ng/ml | Predicted values, ng/ml | Cross-reactivity, % |
|---|---|---|---|
| CEA | 3400 | 1.61 ± 0.31 | 0.047 |
| CA19-9 | 1480 | 1.07 ± 0.25 | 0.072 |
| CA125 | 1200 | 0.78 ± 0.27 | 0.065 |

Note: The cross-reactivity rates of different compounds were all less than 0.10%.

Table S10 Recovery rate of the immunoassay (n=3)

| CEA | | | | AFP | | | |
|---|---|---|---|---|---|---|---|
| Original, ng/ml | Added, ng/ml | Predicted, ng/ml | Recovery, % | Original, ng/ml | Added, ng/ml | Predicted, ng/ml | Recovery, % |
| 21.34 ±1.57 | 19.27 ±1.78 | 41.73 ±2.59 | 105.81 | 50.94 ±4.35 | 32.13 ±3.19 | 81.97 ±3.65 | 96.58 |
| 183.27 ±2.51 | 197.49 ±10.31 | 366.49 ±21.85 | 92.7 | 198.46 ±8.26 | 189.17 ±19.83 | 399.97 ±22.75 | 106.52 |
| 385.39 ±28.43 | 371.22 ±17.85 | 751.88 ±46.29 | 98.73 | 422.98 ±6.41 | 378.34 ±19.11 | 777.66 ±16.31 | 93.75 |
| 554.49 ±35.98 | 172.12 ±7.55 | 737.76 ±38.23 | 106.47 | 554.49 ±35.98 | 172.12 ±7.55 | 737.76 ±38.23 | 106.47 |

Table S11 Harris-Boyd's test for different subgroups

| Subgroup | CEA | | | AFP | | |
|---|---|---|---|---|---|---|
| | z | z* | Partition | z | z* | Partition |
| Female vs Male | 0.70 | 2.67 | No | 2.58 | 2.67 | No |
| ⩽30 vs 30-40 | 2.24 | 2.32 | No | 0.78 | 2.32 | No |
| 30-40 vs ⩾40 | 1.73 | 2.03 | No | 2.75 | 2.03 | Yes |
| ⩽30 vs ⩾40 | 1.01 | 2.17 | No | 1.96 | 2.17 | No |

Note: the z and z* value was calculated by the formula according to CLSI C28-A3 document.
